# Predicting Greater Prairie-Chicken Lek Site Suitability to Inform Conservation Actions

**DOI:** 10.1371/journal.pone.0137021

**Published:** 2015-08-28

**Authors:** Torre J. Hovick, David K. Dahlgren, Monica Papeş, R. Dwayne Elmore, James C. Pitman

**Affiliations:** 1 School of Natural Resource Sciences-Range Program, North Dakota State University, Fargo, ND, United States of America; 2 Department of Wildland Resources, Utah State University, Logan, UT, United States of America; 3 Department of Integrative Biology, Oklahoma State University, Stillwater, OK, United States of America; 4 Department of Natural Resource Ecology and Management, Oklahoma State University, Stillwater, OK, United States of America; 5 Western Association of Fish & Wildlife Agencies, Emporia, KS, United States of America; Institute of Agronomy, University of Lisbon, PORTUGAL

## Abstract

The demands of a growing human population dictates that expansion of energy infrastructure, roads, and other development frequently takes place in native rangelands. Particularly, transmission lines and roads commonly divide rural landscapes and increase fragmentation. This has direct and indirect consequences on native wildlife that can be mitigated through thoughtful planning and proactive approaches to identifying areas of high conservation priority. We used nine years (2003–2011) of Greater Prairie-Chicken (*Tympanuchus cupido*) lek locations totaling 870 unique leks sites in Kansas and seven geographic information system (GIS) layers describing land cover, topography, and anthropogenic structures to model habitat suitability across the state. The models obtained had low omission rates (<0.18) and high area under the curve scores (AUC >0.81), indicating high model performance and reliability of predicted habitat suitability for Greater Prairie-Chickens. We found that elevation was the most influential in predicting lek locations, contributing three times more predictive power than any other variable. However, models were improved by the addition of land cover and anthropogenic features (transmission lines, roads, and oil and gas structures). Overall, our analysis provides a hierarchal understanding of Greater Prairie-Chicken habitat suitability that is broadly based on geomorphological features followed by land cover suitability. We found that when land features and vegetation cover are suitable for Greater Prairie-Chickens, fragmentation by anthropogenic sources such as roadways and transmission lines are a concern. Therefore, it is our recommendation that future human development in Kansas avoid areas that our models identified as highly suitable for Greater Prairie-Chickens and focus development on land cover types that are of lower conservation concern.

## Introduction

Spatially-explicit and empirically derived species distribution models (SDMs) based on ecological niche modeling have changed our ability to manage and plan for conservation in a human modified world [1]. Such models have been used in many ways, including assessing species invasions [2], supporting reserve selection [3], and evaluating the impacts of climate and land use factors on species distributions [4]. Additionally, models that incorporate land use and anthropogenic development can inform planning and monitoring efforts for imperiled species. By understanding the factors that constrain species’ distribution at landscape scales, researchers can create models with known location data and maps that depict the probability of use which can aid conservation planning and actions [5].

As energy demands increase and development of energy infrastructure expands into native rangelands, it is increasingly important to understand the effects of these new sources of fragmentation on native wildlife. Anthropogenic development can negatively affect wildlife species through the reduction and fragmentation of habitat [6], displacement [7, 8], and direct mortality [9], all of which can contribute to population declines. Global demand for energy is projected to increase by 40% in the next 20 years [10], and production of alternative energy and related development are expected to impact 200,000 km^2^ of new land in the United States by 2035 [11]. Proactive approaches for the siting of future energy development can reduce the impact on wildlife, ensuring societal benefits while retaining important ecosystem services.

Planning and siting of development is critical in systems that have already been modified, such as grasslands of the central United States. In particular, within the Great Plains, large and unfragmented parcels of prairie are often targeted for energy development because of favorable high wind conditions, underlying geology (oil and gas), and ownership of large parcels that can reduce contract cost and minimize logistical constraints [12]. This raises conservation concerns because the Great Plains harbor some of the last remaining intact tallgrass prairie and is a stronghold for imperiled grassland bird populations [13]. Therefore, development in this region may have greater impacts than if development took place in previously disturbed sites [14]. One example of a species that may be affected is the Greater Prairie-Chicken (*Tympanuchus cupido*), a grassland obligate species experiencing substantial population declines that still persists throughout much of the Great Plains [15–17].

Once common throughout the Great Plains of North America [18], Greater Prairie-Chickens are now restricted to a small fraction of their original distribution [19]. Historically, population reductions have been associated with habitat loss caused by agriculture development [20], but more recently, reductions have been linked to land management practices and increased fragmentation in grassland habitats [17, 21]. To overcome challenges associated with intensive agriculture, recent research has suggested management practices attempting to restore heterogeneity [17, 22]. However, as momentum grows for management aimed at improving conservation and sustainability on rangelands, greater resource demands from a growing population are likely to increase fragmentation to remaining intact grasslands and negate positive effects associated with conservation-minded management practices [23]. Attempts should be made to balance conservation and development.

Natural resource agencies are under increasing pressure to develop scientifically based methods to balance human demands and conservation of habitat for grassland species [24]. Many challenges face these agencies, however, such as incomplete data representing most species or lack of presence/absence data at the scale required for state or federal management, even for species with the most detailed and intensive monitoring [24]. The use of SDMs that generate relatively accurate habitat use and habitat suitability maps are needed to inform conservation planning in the face of emerging development in rangelands [25, 26].

We used an SDM, maximum entropy modeling (Maxent), to estimate Greater Prairie-Chicken lek suitability in Kansas, USA. Year round Greater Prairie-Chicken seasonal habitats are closely associated with breeding display grounds which may make information gleaned from lek sites more informative than other habitat use metrics [27, 28]. We based our estimates on a suite of biophysical variables associated with known Greater Prairie-Chicken occurrences and anthropogenic structures associated with avoidance behavior [29, 30]. Our overall goal was to generate a model that identified “hotspots” that will allow for focused conservation efforts in the future and adaptive development that can avoid areas of potential importance for Greater Prairie-Chickens. Suitability maps generated through Maxent can be used in future land acquisition and targeted conservation programs to link areas of high probability of use in order to create large core areas needed for sustainable Greater Prairie-Chicken populations.

## Methods

### Datasets

Occurrence data for Greater Prairie-Chicken lek locations were gathered by Kansas Department of Wildlife, Parks, and Tourism (KDWPT) on annual spring surveys. One, 16 km survey was conducted in each of 36 counties distributed across the Greater Prairie-Chicken range within Kansas. Additionally, opportunistic lek locations were gathered by KDWPT personnel and volunteers. Routes were surveyed from a public road at least twice annually during the peak of lek attendance (typically March 20–April 20) and began thirty minutes prior to sunrise during days of no rain or fog when winds were <19 km/h. Surveys were conducted by listening for Greater Prairie-Chicken vocalizations for three minutes every 1.6 km. Leks were defined as ≥ three males displaying in an area both because this number of individuals is less likely to be a spontaneous displaying event and to maintain continuity with historical lek surveys done in the state. Lek occurrences from 2003 to 2011 were used to coincide with available spatial data in a landscape that has experienced significant changes over the last 25 years. The majority of lek locations have been documented in the last ten years as survey efforts have expanded. Leks that occurred in the same area (within 250 m of a previous year) across multiple years were only used once in analysis as they were considered the same lek.

Land cover data was obtained from the State of Kansas geographic information systems Data Access and Support Center (DASC; http://www.kansasgis.org/) at a 30 m resolution. Land cover was mapped in Kansas in 2005 using Landsat satellite imagery. We used the level IV land cover product which is composed of 24 different land cover classes. Land cover classification accuracy was assessed by the DASC using a stratified sample of >16,000 sites, the United States Department of Agriculture Farm Service Agency Common Land Unit databases, and Kansas GAP vegetation database to ground-truth the accuracy of cropland, grassland, and woodland classes. Photo interpretation of the 2005 national agriculture imagery program images served to ground-truth and assess the accuracy of the urban and water land cover classifications. The overall accuracy for the level IV classification was nearly 75%. Specifically, because we were interested in Greater Prairie-Chickens, which are known to prefer areas with greater grass coverage [15], we were interested in the accuracy of warm and cool season grass classes which were >86% and >67%, respectively (http://www.kansasgis.org/). We resampled the land cover layer to a 250 m resolution using majority rule in the Resample tool of ArcToobox, ArcGIS 10.0 [31] in order to match the grain of other data layers available for analysis.

We also downloaded an elevation layer from the DASC website. National elevation data were produced by the United States Geological Survey in 1999. These data provide a seamless mosaic of elevations at 30 m resolution. After creating a single layer representative of the elevation of Kansas, similarly to the land cover layer, we resampled the elevation layer resolution to 250 m to match other environmental layers. Elevation was then used in Slope tool of ArcToolbox, ArcGIS 10.0 to generate a slope raster layer with values measuring (in degrees, 0 to 90) the maximum change in elevation between a target cell and its eight neighbor cells.

As an additional estimate of general vegetation types, we used Moderate Resolution Imaging Spectoradiometer (MODIS) derived phenology products for the State of Kansas. MODIS satellite data was acquired from the Land Processes and Distributed Active Archive Center (https://lpdaac.usgs.gov/lpdaac/products/modis_products_table) at a 250 m resolution for mid-March through mid-May in 2005 because this period best coincides with lek attendance of Greater Prairie-Chickens and matches the timing of the level IV land cover environmental layer. The MODIS Normalized Difference Vegetation Index (NDVI) is designed to provide spatial and temporal comparisons of vegetation phenology using blue, red, and near-infrared reflectance. Vegetation differences are inferred from NDVI by graphically interpreting whether the target contains live green vegetation from the remotely sensed light reflectance.

Finally, we used three anthropogenic layers in models estimating suitable lek habitat because anthropogenic structures have been shown to influence prairie grouse habitat use [29, 30, 32–34]. The three layers represented all roads in Kansas, as summarized in the 2014 U.S. Census Bureau TIGER/Line Shapefile Release, electric transmission lines, a layer maintained by Kansas Corporation Commission, and oil and gas wells, a layer generated by Kansas Geological Survey from data submitted to the Kansas Corporation Commission. Similar to other layers, these anthropogenic layers were publicly available at DASC (http://www.kansasgis.org/), however, the original format, vector polyline shapefile for roads and electric transmission lines and vector point shapefile for oil and gas wells, required further processing to generate raster layers compatible with the modeling algorithm. We used the Fishnet tool of ArcToolbox in ArcGIS 10.0 to generate a polygon shapefile with grid cells of 250 m and calculate the density of the linear features (roads and transmission lines, separately) in each cell. The layers were projected to Albers equal area projection before performing the density calculations, to ensure accurate segment length measurement. Since the electric transmission lines are concentrated in certain regions of the state, we also calculated distance to nearest transmission line using the Near tool of ArcToolbox, ArcGIS 10.0. The final transmission raster layer used in the suitability models contained values of distance to nearest transmission line (for cells without lines) and values of density of transmission lines, rescaled using a 10^6^ factor to separate them from distance values. To calculate density of oil and gas wells in each 250 m cell, we selected the wells with spud date (i.e., well becoming functional) between 2003 and 2011, to match the temporal extent of our lek data. In the Density tool of ArcToolbox, ArcGIS 10.0, we set the search radius to 3x3 cells (as opposed to single cell for roads and transmission lines) since the wells were represented by point features. We did not use wind turbine structures in our analysis because the data layer was not publicly accessible at a sufficient scale for this study, but this does not undermine the importance of incorporating additional layers as they become available.

### Model Development

We used Maxent modeling software version 3.3.3k to examine Greater Prairie-Chicken lek site suitability in Kansas [35]. Maxent requires locations for known species presence and does not require known “absence” data to generate models, as it compares the environmental conditions of presences with 10,000 randomly selected pixels from the background. Additionally, Maxent has been validated over a wide range of species and environmental conditions [36].

We developed four models to examine the influence of environmental variables on the variation of lek site suitability predictions. The first model included all seven data layers (Elevation, slope, land cover, NDVI, density of transmission lines, density of roads, and density of oil and gas wells) and is referred to as the global model. The second, environmental model, focused on the biophysical environmental layers only (Elevation, slope, land cover, and NDVI) and did not include the anthropogenic layers. The final two models only examined one of the two vegetation-related layers in addition to anthropogenic layers (hereafter anthropogenic1-land cover model and anthropogenic2-NDVI model). Our reasoning for creating models with small sets of variables was based on our knowledge and understanding of prairie grouse behavior from previously published work [29, 30, 32–34], and the need for our models to be relatable and simple enough for interpretation to be useful to a broad suite of stakeholders.

All modeling was done at the extent of the entire state of Kansas. We chose this extent for several reasons. First, geopolitical boundaries are often the level that reflects management practices and this was the extent of much of the environmental data layers. Second, Greater Prairie-Chicken distribution covers nearly all of the state of Kansas, with the exception of the southwestern quarter of the state, so use of the entire state falls within the functional niche of Greater Prairie-Chicken. Finally, development decisions will largely be done at the state level, so having suitability maps that mirror this scale will aid in decision making and mitigation.

We evaluated our models using three analyses: the area under the curve (AUC) of the receiver operating characteristic (ROC), the omission error, and one-tailed binomial test. The AUC measures the probability that a randomly chosen presence site will be ranked above a randomly chosen absence site [37]. Because Maxent does not use field-collected absence data, “pseudo-absence” or background points are selected uniformly and at random from the study area during modeling. A random site has an average AUC score of 0.5 while a perfect ranking AUC score for a model is 1.0, and models with scores above 0.75 are considered useful [38]. The ROC test was developed for clinical studies and its value and applicability to evaluating ecological niche models or species distribution models has been questioned, with modifications proposed to address some of the issues raised [37, 38]. However, the ROC AUC is still largely used in the literature, as it is a threshold-independent evaluation method. Here, we report the AUC values but use an additional, threshold-dependent, measure of model performance, the omission error rate (or false negative rate), which represents the proportion of known presence records that are incorrectly predicted absent by the model [39]. To obtain omission error rate, the Maxent model output containing suitability values from 0 to 1 is converted to a binary prediction (present-absent). The conversion requires a suitability threshold value above which all pixels are classified as present and below which all pixels are classified as absent. We used a threshold value that corresponded with a Maxent suitability of 90% of training presences predict19ed present. Thus, we allowed the model prediction to omit 10% of the training presence records to account for possible errors in the training data. We used the thresholded presence-absence predictions to calculate testing omission error rate, or the proportion of presences in the testing dataset that were associated with absence pixels. The third analysis, the one-tailed binomial test, evaluated whether the models predicted present testing lek locations better than a random prediction with the same proportion of area predicted present as the model evaluated [35]. We split our known lek locations into 50% for training and 50% for testing for the model accuracy tests [35]. Finally, we produced a model consensus map by summing the four Maxent model outputs converted to binary, presence-absence predictions.

## Results

We used 870 unique lek locations from spring lek surveys conducted in Kansas from 2003–2011. All models had similar outcomes, with comparable AUC values, all above 0.75, indicating that the models were reliable (Table 1). In addition, the test omission error rates were low among the four models (0.14–0.18), with the lowest omission rate obtained for the environmental model (0.14; Table 1). Binomial tests performed in Maxent for all four models were significant (p < 0.05), indicating that the predictions were better than a random, null model.

**Table 1:** Evaluation of maximum entropy models. Models were generated using 870 unique lek locations from the state of Kansas from 2003–2011. For data layers included in models please see contributions of predictor variables in Table 2.

Model	Training AUC	Test AUC	Test omission rate
Global	0.872	0.829	0.18
Environmental	0.853	0.824	0.14
Anthropogenic1-land cover	0.861	0.818	0.16
Anthropogenic2-NDVI	0.864	0.828	0.17

Note: AUC: area under the curve; NDVI: normalized difference vegetation index.

The best predictor of lek site suitability in all models was elevation (Elevation; range = 204–1,230 m), explaining nearly three times more variation than the second best predictor variable (Table 2). The land cover predictor variable was the second most influential and explained more variation than the NDVI variable, but when land cover was excluded from models (i.e., anthropogenic2-NDVI model), the NDVI variable was ranked second in contribution to the model. Transmission line density was the third most influential parameter and did better at explaining habitat suitability than density of roads or wells (Table 2). The contribution of the slope variable to the models was minimal (<2.5%).

**Table 2:** Percent contribution of predictor variables to four models developed for the Greater Prairie-Chicken. Models were generated using know lek site location data for Kansas, USA, from 2003–2011.

			Parameters				
Model	Elev.	Slope	Land Cover	NDVI	Transmission Line Density	Road Density	Oil and Gas Well Density
Global	55.7	2.4	19.6	11.9	7.9	0.9	1.6
Environmental	62.7	2.5	21.2	13.6	-	-	-
Anthropogenic1-land cover	60.8	2.2	24.8	-	8.8	1.7	1.7
Anthropogenic2-NDVI	66.7	2.3	-	19.5	8.7	1.3	1.5
Mean contribution	61.5	2.3	21.9	15	8.5	1.3	1.6

Note: Elev.: elevation layer; NDVI: normalized difference vegetation index.

**Fig 1:**
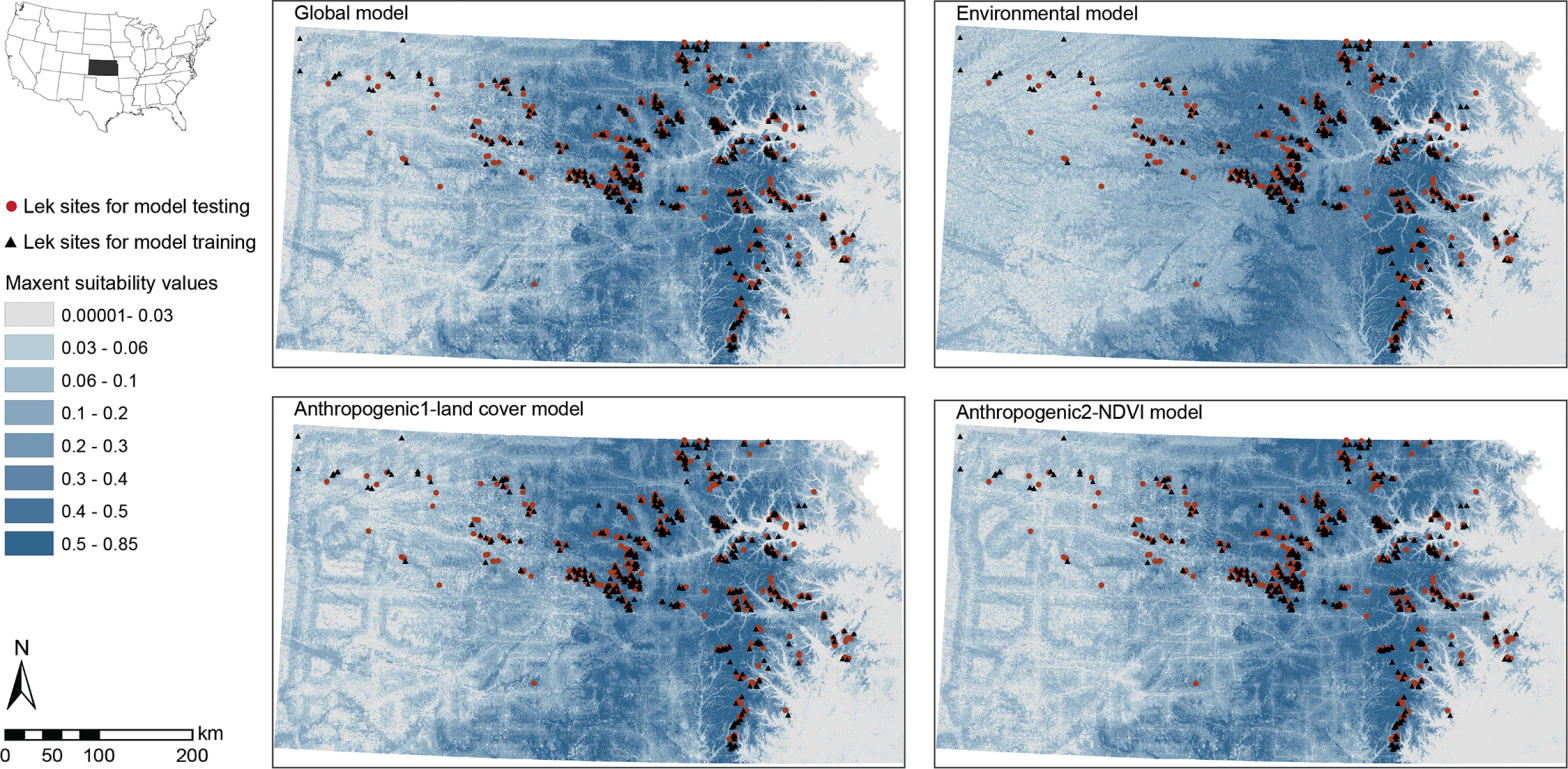
Maps of suitability for Greater Prairie-Chicken in Kansas, estimated using four separate models. Models generated using locations of 870 unique lek sites in Kansas from 2003–2011 (training dataset represented by black triangles and testing dataset by red dots). Areas most suitable are represented by the darkest shade of blue and the lowest by the lightest shade. The global model incorporated elevation, slope, land cover, normalized difference vegetation index (NDVI), density of transmission lines, density of roads, and density of oil and gas wells; the environmental model incorporated elevation, slope, land cover, and NDVI; the anthropogenic1-land cover model included all variables except NDVI and anthropogenic2-NDVI model incorporated all variables except land cover.

**Fig 2:**
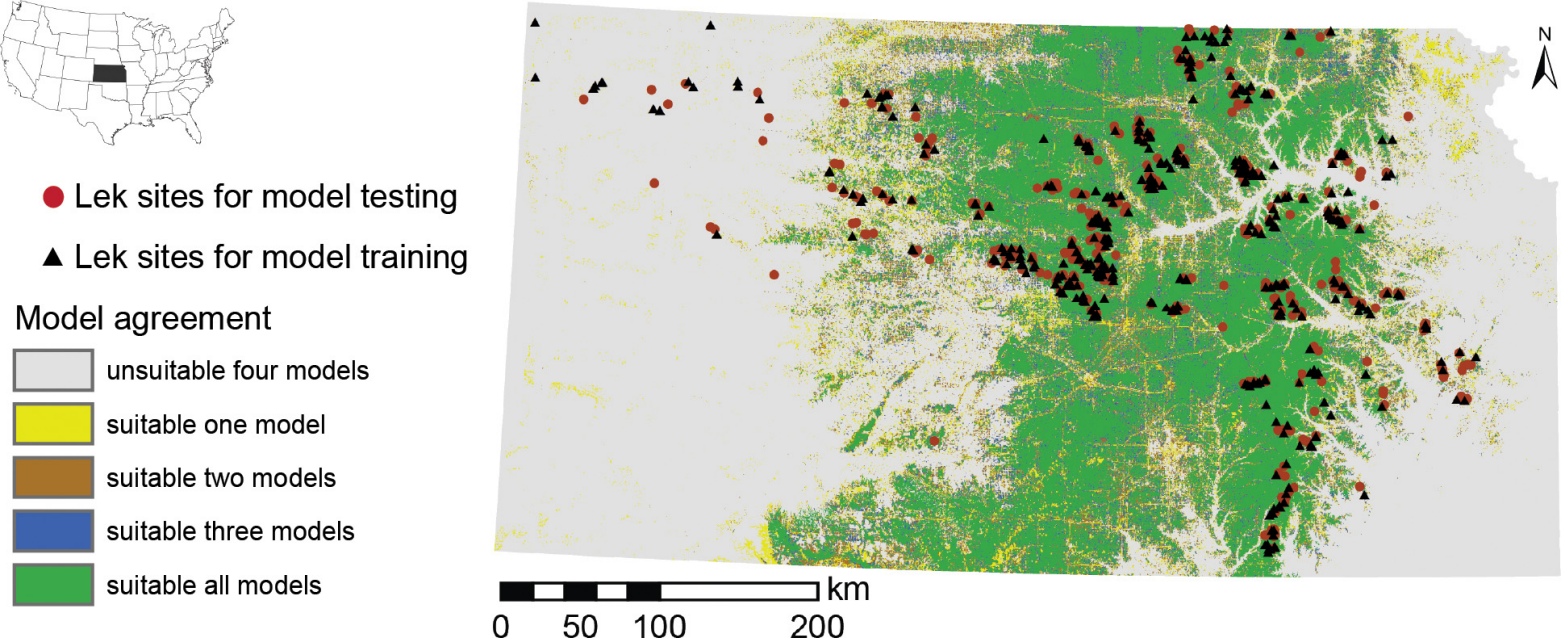
Model consensus map of suitability for Greater Prairie-Chicken in Kansas. Map constructed by summing the four individual models (see Fig 1). The highest model consensus for presence (four models) is shown in green and the lowest (one model) in yellow. Areas predicted absent by all four models are shown in gray. The 870 unique lek sites in Kansas from 2003–2011 are separated in training dataset (black triangles) and testing dataset (red dots).

We generated a distribution map for each model that depicts a range of suitability for lek sites across the entire state of Kansas (Fig 1). Models incorporated various combinations of the seven environmental layers used. Models demonstrated high lek suitability in areas where leks have been detected (Fig 1). However, there are large expanses of predicted “high” quality habitat where lek locations are unknown due to a lack of surveys. The model consensus map (the four models, summed) identified areas that were predicted present by one to four models (low to high model consensus of suitability, respectively) and areas that were predicted absent by all four models (Fig 2).

## Discussion

We found that landscapes most suitable for Greater Prairie-Chicken lek sites are of relatively higher elevation, consisting of grassland vegetation, and low densities of anthropogenic structures. Elevation is a fixed topographic feature and is not a limiting factor to lek site selection where Greater Prairie-Chickens occur in Kansas. However, both vegetation cover, including land cover type, and human development (roads, transmission lines, and oil and gas structures) can be modified to benefit Greater Prairie-Chicken lek site suitability. The products of this analysis will be useful for future conservation actions and are concurrent with other findings focusing primarily on Lesser Prairie-Chicken (*Tympanuchus pallidicinctus*) lek site suitability in the state of Kansas [24].

Greater Prairie-Chickens have evolved in treeless landscapes where predators are commonly aerial, so it is not surprising that this species selects lekking locations that are relatively higher than the surrounding landscape [40]. Selection of higher sites allows individuals to detect predators more easily and is an evolutionary response to increase the vigilance of prairie grouse for predators [41]. Additionally, seeking higher sites relative to the surrounding landscape may have acoustic advantages that allow males to be heard over greater distances but research investigating the acoustics of leks is limited.

From a hierarchical perspective, topographic position explained most of the variation in lek locations. However, in addition to higher elevation, lek sites were in areas that were treeless and composed of grassland vegetation. Previous research points to nesting habitat as the key factor driving lek site selection and this is likely why leks are typically in treeless areas, as Greater Prairie-Chickens select treeless landscapes for nesting [15]. Because males seek to attract females, lek sites are usually near areas where suitable nesting cover exists, which helps explain why 95% of nests were found within 2 km of lek locations in an Oklahoma population of Greater Prairie-Chickens [15, 20]. This phenomenon is commonly referred to as the hot-spot hypothesis, the idea that males display in areas that maximize the potential for them to encounter females [28]. Based on our results, management practices focused on maintaining grassland vegetation while reducing the threat from woody encroachment are most likely to benefit Greater Prairie-Chicken lek site suitability.

In terms of model prediction, anthropogenic structures were not in the most informative model. However, all three models containing anthropogenic structures were considered useful (AUC >0.75). These findings are consistent with other work examining lek site suitability in Kansas that found roadways and oil and gas infrastructure improved model performance and indicated habitat less likely to be utilized be Lesser Prairie-Chickens [24]. Other grouse species are reported to avoid roadways and transmission lines [29, 42], and recent research suggests that wind energy may disrupt habitat use of Greater Prairie-Chickens during certain times of the year, particularly lekking [34, 43]. Generally, grouse species appear sensitive to anthropogenic development and caution should be used when siting new development [30], especially given the availability of area classified as low quality prairie grouse habitat [23].

As with any landscape modeling approaches, there are caveats that need to be considered. First, lek locations were not detected at random and the representation of lek locations used in this model does not depict all potential areas that leks could occur. Surveys were conducted off of roadways and as a result could be biased because the distribution of roadways are not random and the distance from roadways that leks can be detected may be limited by certain features. That said, the density of roads in Kansas and the distance that the sound of booming Greater Prairie-Chickens can be heard reduces the biases that may be associated with lek detection in this dataset and likely makes our results applicable to many similar, agriculture landscapes throughout the Great Plains. Second, lek suitability maps presented here only account for the influence of the biophysical and anthropogenic features that we incorporated in models. Undoubtedly, there are other factors that influence lek locations, including biotic interactions that we could not include in our Maxent models, such as proximity to suitable breeding habitats or interactions with other species. Also, it should be noted that lek sites are treated as presences and our models do not account for the number of birds attending leks, nor do they infer whether locations are representative of increasing or decreasing populations.

Future industrialization of rangelands as a result of human energy demands presents a major conservation concern across the grasslands of the Great Plains and throughout the world [23, 24]. Grasslands in the Great Plains represent one of the largest intact prairie ecosystems remaining in the United States and are stronghold for declining grassland birds [13]. If developers and land managers wish to minimize negative impacts to species of conservation concern, the use of tools such as the suitability maps we created can aid in planning and conservation actions. Generating a habitat suitability map that can be used for future planning of development that may impact wildlife is highly practical and has many applications for managing threatened populations. Our models represent a useful tool for ecologists and land managers alike and further emphasizes that energy and developmental goals can be achieved while avoiding high quality habitat.
